# Antimicrobial Resistance, Biocide Tolerance, and Bacterial Diversity of a Dressing Made from Coriander and Parsley after Application of Treatments Using High Hydrostatic Pressure Alone or in Combination with Moderate Heat

**DOI:** 10.3390/foods11172603

**Published:** 2022-08-27

**Authors:** Javier Rodríguez López, Maria José Grande Burgos, Rubén Pérez Pulido, Belén Iglesias Valenzuela, Antonio Gálvez, Rosario Lucas

**Affiliations:** Microbiology Division, Department of Health Sciences, Faculty of Experimental Sciences, University of Jaén, 23071 Jaén, Spain

**Keywords:** dressing, antimicrobial resistance, high-hydrostatic pressure processing, bacterial diversity

## Abstract

The effects of high-hydrostatic pressure (HP) treatments (450 and 600 megapascals, MPa, for 5 min at temperatures of 22 °C and 50 °C) on the microbiota of a coriander and parsley dressing was studied via culture-dependent and culture-independent approaches. Samples were refrigerated for 20 days, with periodic counts of the culture media supplemented with, or without, antimicrobials. HP-treated samples showed significantly lower viable cell counts compared to untreated controls. Only the control samples yielded bacterial growth on media with antimicrobials (imipenem, cefotaxime, benzalkonium chloride), including mostly *Pseudomonas* and *Lactobacillus*. *Bacillus* and *Paenibacillus* were identified from pressurized samples. Few isolates showed higher tolerance to some of the biocides tested. Pseudomonads showed outstanding resistance to meropenem and ceftazidime. According to high-throughput sequencing analysis, the microbiota of the dressing control samples changes during storage, with a reduction in the relative abundance of *Proteobacteria* and an increase in *Firmicutes*. The composition of the residual microbiota detected during storage was highly dependent on the pressure applied, and not on the treatment temperature.

## 1. Introduction

Vegetable foods are globally regarded as healthy [1] as well as being an essential component of the human diet [2,3]. In the case of dressings and condiments they are often prepared at home or in catering facilities without applying any treatment to inactivate the microorganisms. These ready-to-eat food products may act as vehicles for the transmission of foodborne pathogenic or toxigenic bacteria, and for the spread of antimicrobial resistance [4,5,6], with numerous studies demonstrating this [7,8,9,10]. Herbs used in the preparation of dressings and sauces are often a major source of contamination. Fresh coriander and parsley are often consumed without any treatment to inactivate microorganisms. In addition, they are grown close to the soil, which makes them more susceptible to contamination by irrigation water. In 1995, an outbreak of severe gastroenteritis leading to hemolytic uremic syndrome and thrombocytopenic purpura involved children who had consumed sandwiches that contained parsley contaminated with verotoxigenic *Citrobacter freundii* [11]. Parsley was also associated with outbreaks caused by food contaminated with *Escherichia coli* (ETEC) in restaurants in Minnesota [12]. Other studies have also established a cause–effect relationship between cases of shigellosis and consumption of parsley contaminated with *Shigella sonnei* or *Shigella boydi* [13,14]. Likewise, between 1996 and 2015, the FDA recorded nine outbreaks associated with the consumption of these herbs, with a total of 2699 people affected and 84 hospitalizations [15]. Four of the outbreaks were linked to basil, three to coriander, and two to parsley. Seven of the outbreaks were attributed to the parasite *Cyclospora cayetanensis*, one to *E. coli* O157:H7, and one to *S. sonnei*. In 2021, a *Salmonella* Oranienburg outbreak was reported in the USA, affecting at least 279 people in 29 states according to the Centers for Disease and Prevention [16]. After lengthy investigation, the *Salmonella* strain in question was isolated from a container that had contained a coriander and lime-based condiment [16].

The FDA established, in 2017, a sampling plan for fresh herbs (coriander, parsley, and basil) for the detection of the most important intestinal pathogens (*Salmonella* and Shiga-toxin-producing *E. coli* and the parasite *Cyclospora cayetanensis*), and reported the detection of *Salmonella* and Shiga-toxin-producing *E. coli* in the periodic samplings of domestic and/or imported herbs [17]. As of March 31, 2020, the agency reported that 15 out of the 1272 herb samples collected tested positive for *Salmonella* (six domestic, nine import), and 10 tested positive for Shiga-toxin-producing *E. coli* (five domestic, five import) [17]. Generic *E. coli* was detected in parsley samples from Brazil [18]. The counts of *Enterobacteriaceae* ranged widely from a few samples (3%) with counts of around 2 log CFU per gram, to others (16%) reaching counts of 7–8 log CFU per gram [18]. Other studies have reported initial microbial load values of total aerobic mesophiles between 3.0 and 5.0 log CFU/g for parsley [19], and 6.3 for chopped parsley [20]. For coriander, viable cell counts between 6.7 [21] and 7.0 log CFU/g have been reported [22,23]. In another study, conducted on 132 coriander samples, approximately 90% of the samples were found to have total aerobic counts between 6.00 and 8.99 log CFU/g [24]. Many of the samples also had total coliform concentrations around 3.0 log CFU/g or higher, and *E. coli* was detected in one sample [24]. Antibiotic-resistant *E. coli* from coriander has been reported in Germany [25]. 

The application of non-heat treatments, which allow us to maintain the nutritional and organoleptic properties of the product, but at the same time, ensure the reduction in microbial load and the concentration of resistant microorganisms present in the food, is essential to improve product safety and shelf life [26]. Among these types of techniques applied to food, high-hydrostatic pressure (HP) treatments stand out as preservation methods, and their application has experienced exponential growth in the last decade [27,28,29,30,31]. Previous studies have shown that the efficacy of such treatments with high pressures can be increased in combination with moderate heat [32]. Improving the efficiency of HP treatments may be essential to decrease the microbial load in treated samples. In addition, characterization of the surviving microbiota would allow us to estimate the changes in the microbial populations of the food under study that occur after the application of HP treatments, and to gain information on possible changes in the surviving populations during storage. This issue has hardly been investigated.

A recent study reported that HP processing increased tetracycline resistance as well as the expression of *tet*M gene, and decreased resistance to gentamicin and kanamycin and the expression of the corresponding *aac(6′)-Ie-aph(2″)-Ia* and *aph(3′)-IIIa* genes among strains from commercial starter and protective cultures [33]. The aim of the present work was to study changes in microbial load, bacterial diversity, and antimicrobial resistance in dressing samples treated with HP and stored under refrigeration. To investigate how HP treatments could influence the levels of antimicrobial resistance in the dressing, we chose cefotaxime and imipenem as representatives of third-generation cephalosporins and carbapenems, respectively, and benzalkonium chloride as a representative of quaternary ammonium compounds.

## 2. Materials and Methods

### 2.1. Preparation of Dressing Samples

Coriander and parsley were obtained from five different local stores and supermarkets in the province of Jaén (Spain) the day before sample preparation, and were stored under refrigeration until use. The herbs were cut into small pieces with sterile scissors under aseptic conditions. Freshly made dressing was prepared by directly mixing, with a blender, the following ingredients in the proportions indicated in parentheses (by weight): chopped coriander and parsley (30% each), extra virgin olive oil (30%), water (8.2%), fresh-made lemon juice (1.0%), and salt (0.8%). Duplicate batches (10 g per bag) of the dressing were packed in polyethylene–polyamide bags right before treatments. 

### 2.2. High-Hydrostatic Pressure Treatments

Treatments with high hydrostatic pressure (HP) were applied as described in a previous study carried out on guacamole [34], using Stansted Fluid Power LTD HP equipment (SFP, Essex, UK) with water containing 10% propylenglycol as the pressurization fluid. The system included an electrical heating unit (SFP), which allowed us to apply the following treatment conditions at a gradually increasing speed up to 75 MPa/min followed by almost instant decompression: (A) 450 MPa at 22 °C; (B) 450 MPa at 50 °C; (C) 600 MPa at 22 °C; and (D) 600 MPa at 50 °C. After application of treatments, samples were cooled on ice for 30 min. Control samples were not treated with HP. All samples were stored at 4 °C for up to 20 days.

### 2.3. Microbiological Analysis

The procedures used for the microbiological analysis were the same as described in a previous study [34]. Controls and HP-treated batches (two bags each) were analyzed following HP treatments (time 0) and during the refrigerated storage period (days 2, 5, 10, and 20). Each sample was homogenized with 20 mL sterile saline solution for 1 min (Stomacher 400, Seward, UK) and the pH was measured (Crison Instruments, S.A., Barcelona, Spain). Serial dilutions from homogenates were spread-plated in triplicate using the following media and incubation conditions: trypticase soya agar (TSA; Scharlab, Sentmenat, Spain) for total aerobic mesophiles (37 °C, 24 h), MacConkey agar (Scharlab) for *Enterobacteriaceae* (37 °C, 24 h), and yeast glucose agar plus 100 mg/L chloramphenicol (YGC; Sigma Aldrich, Madrid) for yeasts and molds (48 h, 28 °C.) The serial dilutions were also plated on media containing antimicrobials: MacConkey agar (Scharlab) plus 64 mg/L cefotaxime (Laboratorios Normon, Madrid) or 4 mg/L imipenem (Aurovitas, Madrid, Spain) (both incubated at 37 °C for 24 h); Klebsiella pneumoniae carbapenemase (KPC) agar plus supplement (Sigma) (incubated in duplicate under aerobiosis and anaerobiosis at 37 °C for 24 h); Mueller–Hinton agar (Scharlab) plus 200 mg/L benzalkonium chloride (Sigma-Aldrich) (incubation at 30 °C under anaerobiosis for 24 h). Viable cell concentrations were calculated as log**_10_** colony-forming units, CFU, per gram of sample. Incubation under anaerobic conditions was conducted in anaerobic jars with the Anaerocult™ A system (Merck, Darmstadt, Germany).

A collection of bacterial isolates was prepared as follows. Individual colonies from non-selective and also from selective media were repurified by further dissemination and incubation with the same medium. Well-isolated colonies were subcultured on TSB and stored at −80 °C in TSB containing 20% glycerol.

### 2.4. Identification of Bacterial Isolates

Total DNA of bacterial isolates was extracted using the GenElute™ kit (Sigma-Aldrich, Burlington, MA, USA). Subsequently, the almost-complete 16S ribosomal gene was amplified by polymerase chain reaction (PCR) using the primers 27F (5′-GAG TTT GATCMTGG CTC AG-3′) and 1492R (5′-ACGGYT ACC TTG TTA CGA CTT-3) [35], and subsequently sequenced. The obtained sequences were analyzed with the BLAST algorithm of the National Centre for Biotechnology Information (NCBI, Bethesda, MA, USA).

### 2.5. Determination of Biocide Tolerance

The biocides benzalkonium chloride (BC), hexadecylpyridinium chloride (HDP), cetrimide (CT), triclosan (TC), hexachlorophene (CF), and chlorhexidine (CH) were from Sigma-Aldrich (Madrid, Spain). TC and CF were dissolved (10% *w*/*v*) in 96% ethanol. HDP (5% *w*/*v*) and CT (10% *w*/*v*) were dissolved aseptically in sterile distilled water. CH was dissolved directly in TSB (2% *w*/*v*). Biocide solutions were stored at 4 °C for ≤7 days. Poly-(hexamethylen guanidinium) hydrochloride (PHMG) solution (7.8% of PHMG, *w*/*v*) was a gift from Oy Soft Protector Ltd. (Espoo, Finland). Biocide sensitivity was determined by the broth microdilution method on 96-well flat-bottom microtiter plates (Becton Dickinson Labware, Franklin Lakes, NJ, USA) as described elsewhere [36]. Briefly, TSB supplemented with different biocide concentrations was inoculated (1%, *v*/*v*) with overnight cultures of the bacterial strains in TSB, and then distributed on microtiter plates (200 μL per well). Biocide dilutions in TSB without inoculation were used as negative controls. TSB samples without biocides inoculated with each strain were used as positive controls. Microtiter plates were incubated at 37 °C for 24–48 h before recording the optical density values at 595 nm (iMarkMicroplate Reader, BioRad, Madrid, Spain). All assays were carried out in triplicate, and the coincident results reported as the minimum inhibitory concentration (MIC).

### 2.6. Antimicrobial Resistance Testing

Isolates were tested for antimicrobial sensitivity by the disk sensitivity test on Mueller–Hinton agar (Sigma) following the CLSI guidelines [37]. The antimicrobials tested were amoxicillin–clavulanic acid (AMC, 30 μg), cefoxitin (FOX, 30 μg), cefotaxime (CTX, 30 μg), ceftazidime (CAZ, 30 μg), meropenem (MEM, 10 μg), ciprofloxacin (CIP, 5 μg), gentamicin (CN, 10 μg), kanamycin (K, 30 μg), erythromycin (E, 30 µg), chloramphenicol (C, 30 μg), tetracycline (TE, 30 μg), and sulfonamide (S3, 300 μg). Antibiotic disks were purchased from Oxoid (Basingstoke, UK).

### 2.7. DNA Extraction from Samples

Microbial DNA was extracted as described in a previous study [34]. The pellets recovered from samples (5 mL) after centrifugation (13,500× *g* for 5 min) were resuspended in sterile saline solution (0.5 mL each) and treated with propidium monoazide (PMA™, GenIUL, S.L, Barcelona, Spain) [38,39]. Then, total DNA was extracted (DNeasy PowerSoil Kit; Quiagen, Madrid). Extracted DNA corresponding to the two biological replicates of the same sampling time was pooled into a single sample. The DNA quality and concentration were determined (QuantiFluor^®^ ONE dsDNA system; Promega, Madison, WI, USA).

### 2.8. DNA Sequencing and Analysis

The following standard procedure was used for DNA sequencing and analysis [34]. Amplification of the 16S rDNA V3–V4 regions was performed using the Illumina Metagenomic Sequencing Library Preparation protocol (Illumina, Inc., San Diego, CA, USA) with the primers (F)5′TCGTCGGCAGCGTCAGATGTGTATAAGAGACAGCCTACGGGNGGCWGCAG and (R)5′GTCTCGTGGGCTCGGAGATGTGTATAAGAGACAGGACTACHVGGGTATCTAATCC [40]. After quality assessment with prinseq-lite [41], analysis of the sequence data was performed with qiime2 pipeline [42]. Denoising, joining of paired-end reads, and depletion of chimeras was carried out with the DADA2 pipeline [43]. The naive Bayesian classifier (integrated into qiime2 plugins) and the SILVA_release_132 database were used for taxonomic affiliation [44]. 

### 2.9. Statistical Analysis

Data were analyzed using one-way ANOVA and Tukey’s tests, as well as principal coordinates analysis.

## 3. Results

### 3.1. Influence of Pressure Treatments and Storage Time on pH, Microbial Load, and Antimicrobial-Resistant Populations

The pH of control samples (not treated with HP) decreased significantly from 5.06 at time 0 to 4.66 at day 10, followed by an increase at day 20 (Table 1). By contrast, pH values of HP-treated samples remained very stable during storage (between 4.94 and 5.11), with no significant (*p* > 0.05) differences between samples (regardless of treatment or storage time).

The viable cell counts of total aerobic mesophiles in the control samples increased gradually and significantly (*p* < 0.05) from 5.49 to 6.58 log CFU/g during storage. Samples treated with HP showed viable cell counts comprised between 1.02 and 1.84 log CFU/g that were significantly lower (*p* < 0.05) compared to untreated controls, or even were below the detection limit of 1.0 log CFU/g (as in the case of three samples pressurized at 50 °C; Table 1). Given the low residual counts obtained for the treated samples, it was not possible to detect statistically significant differences between treatments with regard to pressure or temperature.

Counts of presumptive *Enterobacteriaceae* obtained on MacConkey agar increased from 5.5 to 6.25 at day 10, but still remained significantly lower (*p* < 0.05) compared to total aerobic mesophiles for days 2 to 20. No viable counts were obtained on MacConkey agar for any of the HP-treated samples (Table 1).

Yeasts and molds remained at levels between 5.19 and 5.5 log CFU/g for most of the control samples, with only significant differences for storage time 10. Most of the yeast and mold counts obtained were significantly lower than the total aerobic mesophile count. Most of the counts for yeasts and molds for the HP-treated samples were below the detection limit, except for time 0 in samples pressurized at 450 MPa, 50 °C (Table 1).

To investigate how antimicrobial resistance would be affected by HP treatments, growth on media containing different antimicrobials was determined. Remarkably, no bacterial growth was obtained from any of the samples treated with HP during the complete storage period. In the control samples (not treated with HP), microbial growth was observed for most of the samples after incubation in media with different antimicrobials, but the viable cell counts obtained were in most cases significantly lower compared to counts obtained without antimicrobials, with some exceptions, which will be pointed out below.

For the biocide benzalkonium chloride, viable counts could only be reported for control samples at days 2 and 5 of storage, and they were quite low (1 and 2.3 log CFU/g; Table 2).

Bacterial counts of control samples on MacConkey agar supplemented with cefotaxime ranged from 2.58 to 3.70 log CFU/g (days 2 to 10) or were below detectable levels (day 20; Table 2). Counts for cefotaxime were significantly lower (*p* < 0.05) compared to counts obtained on the same medium without antibiotic. Viable counts on MacConkey agar plus imipenem were high for most of the storage points (4.47 to 5.18 log CFU/g) except day 20 (3.08 log CFU/g) and they were significantly higher (*p* < 0.05) compared to cefotaxime (Table 2). Counts obtained on imipenem for days 2, 5, and 10 did not differ significantly from counts obtained at time 0 on TSA or times 0 and 5 on MacConkey agar without antibiotics. 

Results from KPC agar under aerobic conditions were as high as those obtained on MacConkey agar plus imipenem. As a matter of fact, both sets of counts did not differ significantly for several sampling points. Nevertheless, the ability to grow on KPC agar under anaerobic conditions was markedly reduced. No growth was obtained for storage times 0 and 20, and the viable counts obtained for days 2 to 10 (2.40 to 3.57 log CFU/g) were significantly lower (*p* < 0.05) compared to counts obtained after incubation under aerobiosis on the same medium for the first 10 days of storage (Table 2).

### 3.2. Identification of Bacterial Isolates

A total of 80 bacterial isolates (including 22 isolates grown on TSA from the pressurized samples and 58 isolates from control samples plated on media containing antimicrobials) obtained at different incubation times were studied (Table 3). Most of the isolates were identified as members of the genera *Pseudomonas* (38.75%), *Bacillus* (26.25%), *Paenibacillus* (15.00%), and *Lactobacillus* (12.50%). The rest of the isolates belonged to the genera *Obesumbacterium* (2.5 %), *Rahnella*, *Siccibacter*, *Aerococcus*, and *Staphylococcus* (1.25% each). The main species of *Pseudomonas* detected were *P. lactis* (10 isolates) and *P. paralactis* (eight isolates). The main endospore formers identified were *B. endophyticus* (14 isolates) and *P. xylanilyticus* (eight isolates). Finally, *L. graminis* (nine isolates) was the main representative among lactobacilli.

Isolates obtained from the pressurized samples (on non-selective medium) belonged to the genera *Bacillus* (10 isolates), *Paenibacillus* (11 isolates), and *Aerococcus* (one isolate). The main group of isolates obtained from the control samples on selective media supplemented with antimicrobials was *Pseudomonas* (including all 31 isolates from the collection): 15 isolates from MacConkey agar supplemented with cefotaxime, 12 isolates from MacConkey agar supplemented with imipenem and four isolates from KPC agar incubated under aerobic conditions.

### 3.3. Biocide Tolerance of Isolates

**Figure 1:**
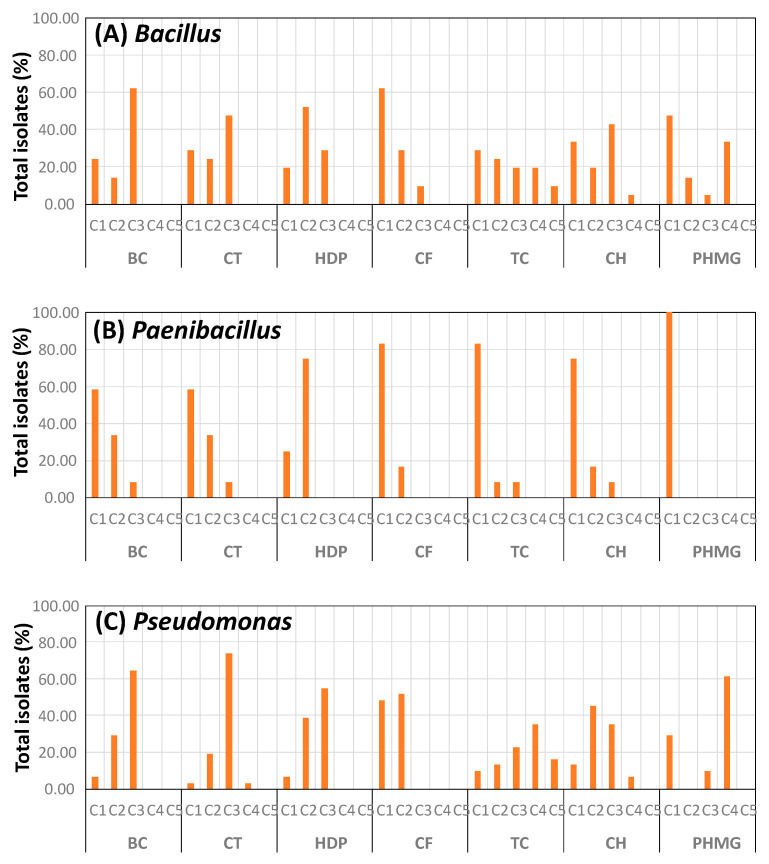
Percentage of isolates for each minimum biocide concentration (MIC). (**A**) *Bacillus*; (**B**) *Paenibacillus*; (**C**) *Pseudomonas*. The biocide concentrations tested were: C1, 2.5 µg/mL; C2, 25.0 µg/mL; C3, 250.0 µg/mL; C4, 2500.0 µg/mL; C5, 10,000.0 µg/mL.

The results of biocide tolerance for the main bacterial groups of isolates are shown in Figure 1. Three patterns of MIC distributions were observed: (1) a high percentage of isolates being inhibited at a given biocide concentration, with no isolates requiring other concentrations higher than this for inhibition. Examples are BC for *Bacillus* and *Pseudomonas*, HDP and PHMG for *Paenibacillus* and *Pseudomonas*, and CF for *Pseudomonas*; (2) most isolates were inhibited at a given biocide concentration, but only a few required a higher concentration for inhibition (supposedly being more biocide tolerant). For example, TC, CF, and CH for *Paenibacillus*, and CT for *Pseudomonas*; (3) a broad distribution of MICs, as in TC for *Bacillus* and *Pseudomonas*, suggesting a broad heterogeneity of the isolates in sensitivity to this biocide.

### 3.4. Antimicrobial Resistance of Pseudomonas Isolates

Results concerning the antimicrobial resistance of the 31 *Pseudomonas* isolates are shown in Table 4. Most or all isolates were resistant to AMC (96.77%), FOX (100%), CTX (100%), and E (87.09%). In addition, resistance was also detected for CAZ (19.35%), MEM (32.26%), K (64.52%), C (25.80%), and S3 (9.68%). All isolates were susceptible to CIP, G, and TE.

### 3.5. Effect of Treatments on the Bacterial Diversity of Samples

The numbers of reads assigned to operational taxonomic units (OTUs) ranged from 35,034 to 62,467 (Supplementary Table S1). Shannon and Simpson diversity indexes were lowest in control samples during storage. Most of the pressurized samples also showed low diversity values after treatment, but not during storage.

**Figure 2:**
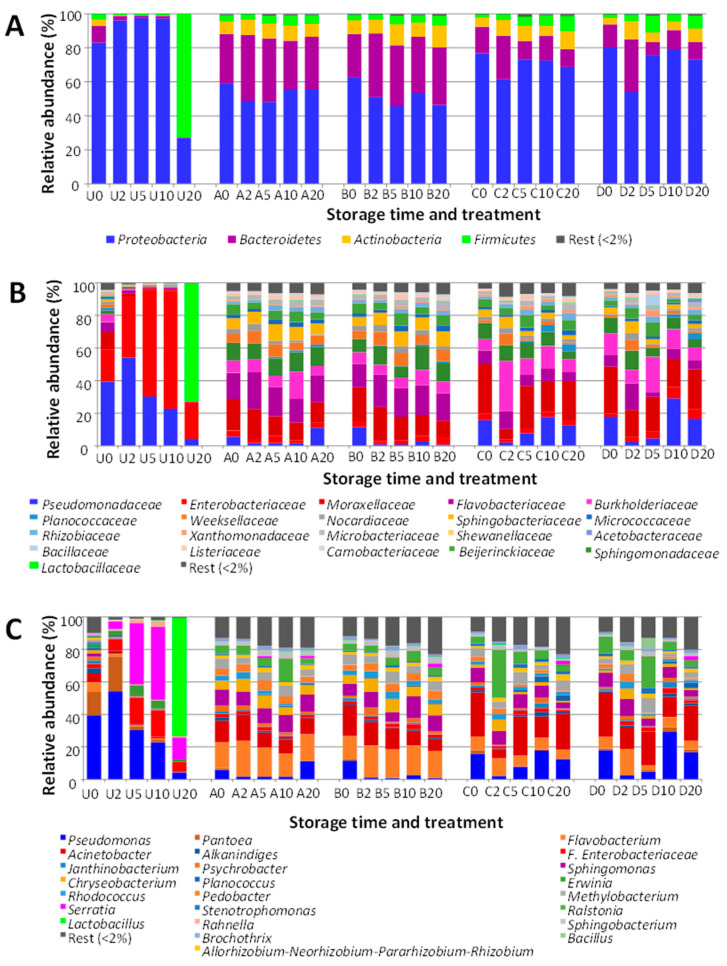
Bacterial diversity of the dressing samples. (**A**) phylum; (**B**) family; (**C**) genus. U, untreated controls. Samples subjected to the different HP treatments are indicated by letters A (450 MPa, 22 °C), B (450 MPa, 50 °C), C (600 MPa, 22 °C), and D (600 MPa, 50 °C). The numbers indicate the storage time (days).

The relative abundance values for the operational taxonomic units (OTUs) from the dressing samples are shown in Figure 2. *Proteobacteria* (83.10%) was the main phylum detected in the control dressing samples (Figure 2A), followed by *Bacteroidetes* (9.95%), *Actinobacteria* (3.85%), and *Firmicutes* (2.88%). During the first 10 days of refrigerated storage, the relative abundance of *Proteobacteria* increased up to 97.61%, while the other groups decreased. At the end of storage (day 20), *Proteobacteria* decreased down to 27.20%, while *Firmicutes* became the phylum with highest relative abundance (72.71%).

*Proteobacteria* in the control dressing were represented mainly by *Gammaproteobacteria* (Fam. *Pseudomonadaceae*, followed by *Enterobacteriaceae* and *Moraxellaceae*; Figure 2B). *Enterobacteriace* was the most abundant group between days 2 and 10. Gen. *Pseudomonas* was the main representative in control dressing at times 0 and 2, decreasing afterwards in relative abundance (Figure 2C). The main representative of Fam. *Enterobacteriaceae* at times 0 and 2 was Gen. *Pantoea*, but later on (especially times 5 and 10) *Serratia* and other unassigned genera were the most abundant OTUs in this group. At the end of the storage period (day 20), the microbiota shifted to *Firmicutes* (Fam. *Lactobacillaceae*), with Gen. *Lactobacillus* as most abundant OTU. Nevertheless, *Serratia* and other *Enterobacteriaceae* were also detected, with low relative abundances.

The high-pressure treatments induced major changes in the microbiota, and the relative abundances of the main groups remained reasonably stable during storage. At the phylum level, there were observable differences between samples pressurized at 450 MPa and those treated at 600 MPa, but not between treatments at 22 °C or at 50 °C. As a general rule, *Proteobacteria* remained the main bacterial group in all treated samples, followed by *Bacteroidetes*, *Actinobacteria*, and *Firmicutes* (Figure 2A). For the lower pressure treatments (450 MPa), *Proteobacteria* had relative abundances in the ranges of 45.74 to 62.59%, and *Bacteroidetes* was the second most abundant group, with relative abundances between 25.48 and 37.44%. In the samples pressurized at 600 MPa, the relative abundances of *Proteobacteria* were slightly higher (54.01 to 80.23%). Statistical analysis of the relative abundances of *Proteobacteria* in the pressurized samples indicated a significantly higher abundance (*p* < 0.05) in the samples pressurized at 600 MPa compared to samples pressurized at 450 MPa. However, for the same pressure treatment, there were no significant differences (*p* > 0.05) for *Proteobacteria* between samples treated at 22 °C and samples treated at 50 °C. The group of *Bacteroidetes* had significantly lower relative abundances (*p* < 0.05) in the samples pressurized at 600 MPa compared to those treated at 450 MPa. 

The relative abundances of the groups of *Proteobacteria* found in the pressurized samples differed greatly from the control samples. In the samples pressurized at 450 MPa, Fams. *Pseudomonadaceae* and *Enterobacteriaceae* had very low relative abundances, while other families of *Proteobacteria* became more noticeable (mainly *Moraxellaceae*, *Sphingomonadaceae*, and *Burkholderiaceae*). *Bacteroidetes* were represented mainly by *Flavobacteriaceae*, *Weeksellaceae*, and *Sphingobacteriaceae*. *Flavobacterium*, *Acinetobacter*, and *Sphingomonas* were the genera with the highest relative abundances in the samples pressurized at 450 MPa.

The higher proportion of *Proteobacteria* detected in the samples pressurized at 600 MPa mainly included *Pseudomonadaceae*, *Moraxellaceae*, *Sphingomonadaceae*, and *Burkholderiaceae*. The low relative abundance of *Bacteroidetes* involved mainly the Fams. *Flavobacteriaceae*, *Sphingobacteriaceae*, and *Weeksellaceae*. These three families had significantly lower relative abundances (*p* < 0.05) in the samples pressurized at 600 MPa compared to 450 MPa, as did their representative genera *Flavobacterium* and *Chryseobacterium*.

The average relative abundances calculated for Gen. *Acinetobacter* in the group of samples pressurized at 450 MPa (11.17% for the samples treated at 22 °C and 12.49% for those pressurized at 50 °C) were lower compared to samples pressurized at 600 MPa (18.27% and 18.91%); however, the differences between the two groups were not statistically significant (*p* > 0.05). The same observation was made for Gen. *Pseudomonas* (with average values of 4.42 and 3.40% in the samples treated at 450 MPa, and 11.18 or 14.14 for the 600 MPa treatments).

**Figure 3:**
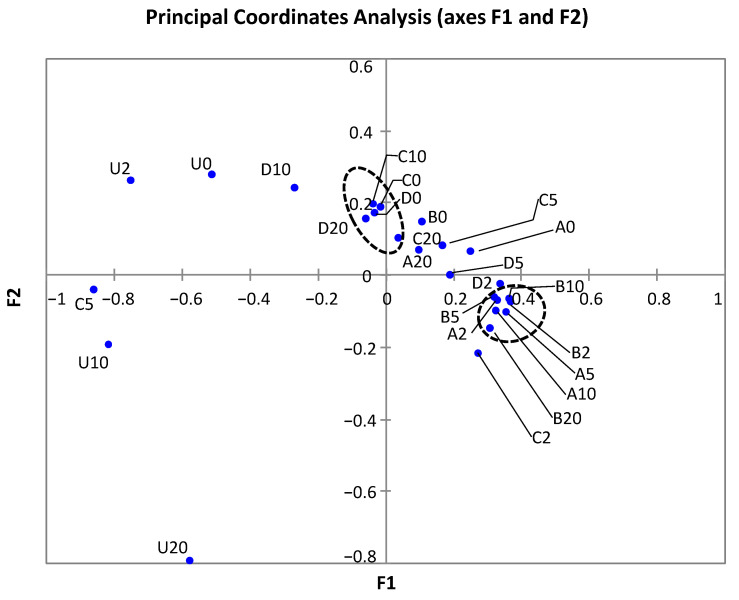
Principal coordinates analysis (PCoA) of dressing samples treated and untreated with HP. U, untreated controls. HP treatments are indicated by letters A (450 MPa, 22 °C), B (450 MPa, 50 °C), C (600 MPa, 22 °C), and D (600 MPa, 50 °C). The numbers indicate the storage time (days).

Multidimensional scaling by principal coordinates analysis (PCoA) indicated an increase in the distance between control samples as incubation time proceeded, which is in agreement with the results obtained on bacterial diversity (Figure 3). Additionally, the pressurized samples mapped separately from the control samples. Most of the samples pressurized at 450 MPa mapped closely and separated from the group of samples pressurized at 600 MPa, although there were some exceptions. These results agree with the differences detected in the proportions of *Proteobacteria* and *Bacteroidetes* and their main representative genera between the two groups of pressurized samples, and also between the pressurized samples and controls.

## 4. Discussion

In the present study, the dressing prepared with coriander and parsley as the main ingredients had a high microbial load for total mesophilic aerobes and *Enterobacteriaceae*, as well as for yeasts and molds, suggesting a low quality of the raw materials. During storage, counts reached levels of 6.25–6.5 log CFU/g, which, in addition to causing product spoilage, could constitute a health risk considering the load of presumptive *Enterobacteriaceae*. The dressing was stable for the first 2 days, but it showed signs of deterioration (off-odor and color change) by day 5. In a previous study carried out by our group with chopped parsley stored in trays, the concentrations of total mesophilic aerobes increased from 6.3 at time 0 to 9.4 log CFU/g after 10 days of storage [20], values that are much higher than those achieved in the present study for the dressing samples packaged in sealed bags. This could indicate that the packaging conditions during storage are important for the preservation of the product. The release of intracellular fluid after chopping could also provide nutrients for the increase in microbial growth. 

Although there are numerous studies on the application of different treatments for the disinfection of vegetable foods, including high pressure (HP), the latter method is rarely applied to products such as dressings and condiments; thus, there are not many works similar to this one. In a study carried out by our group on chopped parsley, the application of HP treatments (600 MPa, 8 min, at 22 °C) reduced the initial microbial load by 3.7 logarithmic cycles, and delayed the growth of the surviving fraction during the 10 days that the treated product was kept under refrigeration, although counts close to 5 log CFU/g were reached at day 10 [20]. The presence of a residual fraction of microorganisms that survive high-pressure treatments can be a problem if they proliferate and reach high concentrations. For this reason, combined treatments with moderate heat and pressure were tested in the present study. Compared to another study on guacamole samples [34], where the same HP treatments were applied, the surviving fraction in dressing was very low, and hardly any proliferation was detected during storage. Thus, the pressurized dressing samples were stable microbiologically, and only exhibited a gradual change in color after day 10 of storage. Most importantly, no viable cells were detected for any of the HP-treated samples in any of the selective media used for presumptive *Enterobacteriaceae*, nor in the antibiotic- or biocide-added media. We should not rule out the presence of sublethally injured cells unable to grow on media with antibiotics. In this case, the results suggest that they would not be able to repair cell damage under acidic conditions (pH close to 5) or during refrigerated storage. This indicates that HP treatments in combination with refrigerated storage under acidic conditions are effective in combating the transmission of antimicrobial resistant bacteria through food. 

Notably, identification of bacterial isolates from the pressurized samples under non-selective conditions revealed mostly *Bacillus endophyticus* and *Paenibacillus xylanilyticus*, two endospore formers associated with vegetables and soil [45,46]. The presence of these bacteria in the residual population after HP treatments could be explained by the higher resistance of bacterial endospores under HP conditions [47]. Most of the bacterial isolates obtained from the control samples on media supplemented with antimicrobials belonged to *Pseudomonas* (*P. lactis* and *P. paralactis*), together with *Lactobacillus graminis* (a species associated with vegetables) [48], and some *Bacillus* and *Paenibacillus*. Members of *Pseudomonas*, *Bacillus*, and *Paenibacillus* are often involved in food spoilage. The presence of these bacterial groups in food processing environments could mean a higher exposure to biocides, and also a higher risk of developing biocide tolerance. The results from the present study indicate that some of the isolates from these bacterial groups showed a higher biocide tolerance than the rest. Therefore, these bacteria could have a selective advantage when exposed to biocides if they enter the food processing environment, and thus, represent a higher risk for contamination and food spoilage.

The bacterial growth obtained from control samples on media supplemented with antimicrobials would suggest intrinsic resistance (as exemplified by *L. graminis* isolates obtained on KPC agar under anaerobiosis) or acquired resistance. Among the main bacterial groups detected, *Pseudomonas* isolates were investigated for antimicrobial resistance because this genus includes representatives of clinical relevance, and also spoilage bacteria that can be present in the food chain. Particularly, *P. lactis* has been described as one of the main groups of spoilage bacteria in raw milk [49]. Most important, the results from the present study indicate that, in addition to what could be intrinsic resistance (AMC, FOX, CTX, E) according to the scientific literature [37], several *Pseudomonas* isolates were also resistant to clinically relevant antibiotics, such as MEM and CAZ. The mechanism(s) of resistance in these isolates, and the risks of transmission of resistance through the food chain, should be further evaluated.

The results obtained in the microbial biodiversity study indicate that the main groups found in the dressing (*Proteobacteria*, followed by *Bacteroidetes*, *Actinobacteria*, and *Firmicutes*) are similar to those described in other vegetable foods, such as guacamole sauce [34] and parsley [20]. However, this composition differs when broken down to lower taxonomic levels, such as family or genus. Important differences are also found in the changes that occur during sample storage. This is a very important aspect for the food industry, since, according to these changes, it is possible to make predictions about the risks that the food may present when it is close to its expiry date, due to the possible presence of spoilage, and pathogenic or toxin-producing bacteria. For example, in the parsley samples, the dynamics of the microbial populations in the control samples were marked by an increase in the proportion of *Bacteroidetes* at the final stages of the storage period, whereas in the cases of both guacamole and dressing, the dynamics were clearly characterized by a considerable increase in *Firmicutes*. It is also interesting to note that the latter were represented in both cases by lactic acid bacteria (mainly *Leuconostocaceae* and *Lactobacillaceae* in guacamole and *Lactobacillaceae* in dressing), indicating that the shelf life of both types of food would be limited by the onset of lactic acid fermentation. This type of change in the microbiota (although it would have undesirable effects on the product) is one that occurs naturally in the fermentation of vegetables, and has also been described in other studies on the storage of vegetable foods, such as cherimoya pulp [50]. However, in the case of the dressing, a significant increase in the relative abundance of *Serratia* was detected in the days prior to the start of lactic acid fermentation. This group of bacteria is important as a pathogen and also as a transmitter of antimicrobial resistance.

The results obtained on the biodiversity of the dressing samples treated with high pressure also show that this type of treatment has a very important effect on the microbiota, as it stabilizes the residual populations, making the changes that occur during storage much smaller compared to the control samples. However, those genera that had higher relative abundances in the control samples (such as *Pseudomonas* or *Serratia*) decreased in the treated samples, while other genera (such as *Flavobacterium* or *Acinetobacter*) showed higher relative abundances. These results can be interpreted considering the differences in sensitivity to high-pressure treatments of each microbial group, as well as differences in their ability to repair sublethal damage caused by treatments under the environmental conditions of the food (pH, nutrients, time, and temperature), and the competition between the different populations. Further studies based on challenge tests will allow a better evaluation of the risks of possible pathogenic or toxin-producing bacteria. It should also be mentioned that, in the pressurized samples, the relative abundance of *Bacillus* was very low, except for sample D5. These results are in contrast with those obtained using the culture-dependent approach, in which the majority of isolates belonged to *Bacillus* and *Paenibacillus*, as discussed above. These results can be explained considering that: (i) most of the endospore formers surviving HP treatment would be in the form of bacterial endospores, with a low or very low yield regarding DNA extraction, and therefore, an apparently low relative abundance in the culture-independent analysis; (ii) most of the non-endospore formers could be sublethally injured and not able to grow on TSA; and (iii) the concentrations of survivors detected were very low, and many of the non-endospore formers detected with the culture-independent approach could be at concentrations below the detection limit. These results reinforce the value of combining culture-dependent and culture-independent analyses on foods.

In conclusion, results from the present study indicate that HP treatments are effective at reducing the microbial load in a dressing prepared from herbs and containing high levels of bacterial contamination, and at the same time, are able to decrease the levels of bacteria resistant to the antibiotics and the biocide investigated. 

## Figures and Tables

**Table 1:** Viable cell concentrations and pH of controls and HP-treated samples.

**Total Aerobic Mesophiles**	**T0**	**T2**	**T5**	**T10**	**T20**
Controls	5.49 ± 0.15	6.13 ± 0.12 ^a^	6.49 ± 0.04 ^a^	6.57 ± 0.15 ^a^	6.50 ± 0.16 ^a^
Treatment A	1.30 ± 0.14 ^b^	1.65 ± 0.21 ^b,d^	1.30 ± 0.04 ^b^	1.84 ± 0.43 b	1.54 ± 0.10 ^b^
Treatment B	1.02 ± 0.07 ^b^	1.39 ± 0.13 ^b^	1.30 ± 0.15 ^b^	1.30 ± 0.11 ^b^	< 1.00
Treatment C	1.02 ± 0.02 ^b^	1.17 ± 0.03 ^b^	1.17 ± 0.15 ^b^	1.74± 0.24 ^b,c,d^	1.77 ± 0.20 ^b,c,d^
Treatment D	1.30 ± 0.22 ^b^	1.54 ± 0.14 ^b^	<1.00	1.17 ± 0.17 ^b^	<1.00
***Enterobacteriaceae***	**T0**	**T2**	**T5**	**T10**	**T20**
Controls	5.50 ± 0.07 ^e^	5.78 ± 0.23 ^e^	5.55 ± 0.22 ^e^	6.25 ± 0.19 ^f^	6.04 ± 0.11
Treatment A	<1.00	<1.00	<1.00	<1.00	<1.00
Treatment B	<1.00	<1.00	<1.00	<1.00	<1.00
Treatment C	<1.00	<1.00	<1.00	<1.00	<1.00
Treatment D	<1.00	<1.00	<1.00	<1.00	<1.00
**Yeasts and Molds**	**T0**	**T2**	**T5**	**T10**	**T20**
Controls	5.19 ± 0.27	5.54 ± 0.10 ^h^	5.17 ± 0.13 ^h^	4.32 ± 0.20 ^g,h^	5.49 ± 0.05 ^h^
Treatment A	<1.00	<1.00	<1.00	<1.00	<1.00
Treatment B	1.01 ± 0.02 ^b^	<1.00	<1.00	<1.00	<1.00
Treatment C	<1.00	<1.00	<1.00	<1.00	<1.00
Treatment D	<1.00	<1.00	<1.00	<1.00	<1.00
**pH**	**T0**	**T2**	**T5**	**T10**	**T20**
Controls	5.06 ± 0.05	4.89 ± 0.05	4.85 ± 0.07	4.66 ± 0.05 ^i^	5.53 ± 0.11 ^j^
Treatment A	5.09 ± 0.08	4.94 ± 0.04	5.00 ± 0.07	4.94 ± 0.08	5.01 ± 0.05
Treatment B	4.98 ± 0.02	5.11 ± 0.08	4.95 ± 0.07	4.96 ± 0.05	4.95 ± 0.08
Treatment C	5.02 ±0. 02	5.03 ± 0.05	5.01 ± 0.04	5.02 ± 0.01	5.01 ± 0.00
Treatment D	4.99 ± 0.01	4.97 ± 0.05	4.98 ± 0.01	4.98 ± 0.02	4.94 ± 0.02

Letters indicate HP treatments. A: 450 MPa, 22 °C. B: 450 MPa, 50 °C. C: 600 MPa, 22 °C. D: 600 MPa, 50 °C. The storage time in days is indicated by (T). Statistical significance (*p* < 0. 05): ^a^, significantly higher than control counts at storage time 0; ^b^, significantly lower than untreated controls (all sampling points); ^c^, significantly higher than same treatment counts at time 0; ^d^, significantly higher than counts at 450 MPa, 50 °C, time 0; ^e^, significantly lower than total aerobic mesophiles at times 5, 10, and 20; ^f^, significantly higher within its group at times 0 and 5; ^g^, significantly lower than the other samples within the group; ^h^, significantly lower than total aerobic mesophiles (times 2 to 20); ^i^, significantly lower pH than all treated samples and also the controls at time 0; ^j^, significantly higher pH than all other samples.

**Table 2:** Viable cell counts of control dressing samples on media with antimicrobials.

Antimicrobial	T0	T2	T5	T10	T20
Cefotaxime	2.57 ± 0.18 ^a^	3.76 ± 0.33 ^a^	3.26 ± 0.09 ^a^	3.70 ± 0.25 ^a^	<1.00
Imipenem	4.47 ± 0.09 ^a,c^	5.15 ± 0.15 ^b,c^	5.17 ± 0.19 ^b,c^	5.12 ± 0.11 ^b,c^	3.07 ± 0.12 ^a^
KPC aerobiosis	5.11 ± 0.10 ^c^	5.20 ± 0.05 ^c^	5.05 ± 0.12 ^c^	4.81 ± 0.22 ^c^	2.88 ± 0.06
KPC anaerobiosis	<1.00	2.39 ± 0.13 ^d^	2.34 ± 0.03 ^d^	3.56 ± 0.18 ^d^	<1.00
Benzalkonium chloride	<1.00	1.02 ± 0.02	2.32 ± 0.21	<1.00	<1.00

KPC, *Klebsiella pneumoniae* carbapenemase agar plus supplement. T, storage time (days). No viable cells were detected in the antimicrobial-supplemented media in the pressurized samples. Statistical significance (*p* < 0.05): ^a^, significantly lower value than counts obtained on MacConkey agar without antimicrobials (based on *Enterobacteriaceae* counts in Table 1); ^b^, significantly lower than counts obtained on MacConkey agar without antimicrobials at times 2, 10, and 20; ^c^, significantly higher than counts obtained on cefotaxime; ^d^, significantly lower compared with KPC counts under aerobiosis at times 0 to 10.

**Table 3:** Identification of bacterial isolates obtained from controls and from pressurized samples.

Genera (nº Isolates; %)	Species	Nº Isolates
*Aerococcus* (*n* = 1; 1.25%)	*A. viridans*	1
*Bacillus* (*n* = 21; 26.25%)	*B. endophyticus*	14
	*B. filamentosus*	1
	*B. oceanisediminis*	2
	*B. safensis*	1
	*B. simplex*	2
	*B. zhangzhouensis*	1
*Lactobacillus* (*n* = 10; 12.50%)	*L. curvatus*	1
	*L. graminis*	9
*Obesumbacterium* (*n* = 2; 2.50%)	*O. proteus*	2
*Paenibacillus* (*n* = 12; 15.00%)	*P. illinoiensis*	1
	*P. taichungensis*	1
	*P. tundrae*	1
	*P. xylanexedens*	1
	*P. xylanilyticus*	8
*Pseudomonas* (*n* = 31; 38.75%)	*P. koreensis*	1
	*P. lactis*	10
	*P. lurida*	1
	*P. paralactis*	18
	*P. trivialis*	1
*Rahnella* (*n* = 1; 1.25%)	*R. aquatilis*	1
*Siccibacter* (*n* = 1; 1.25%)	*S. turicensis*	1
*Staphylococcus* (*n* = 1; 1.25%)	*S. capitis*	1

**Table 4:** Antimicrobial resistance of *Pseudomonas* isolates.

Isolate	Day	Species	Antimicrobial Resistance *
CI21	5	*P. koreensis*	AMC, FOX, CTX, C, K, E
K25	10	*P. lactis*	AMC, FOX, CTX, MEM, E
CF3	0	*P. lactis*	AMC, CAZ, FOX, CTX, S3, E
CF19	5	*P. lactis*	FOX, CTX, E
CF24	5	*P. lactis*	AMC, FOX, CTX, C, E
CF28	10	*P. lactis*	AMC, CAZ, FOX, CTX, C, E
CF30	10	*P. lactis*	AMC, CAZ, FOX, CTX, C, E
CF34	10	*P. lactis*	AMC, FOX, CTX, C
CI19	5	*P. lactis*	AMC, FOX, CTX, S3
CI25	10	*P. lactis*	AMC, FOX, CTX, C, E
CI26	10	*P. lactis*	AMC, FOX, CTX, MEM, K, E
CI33	10	*P. lurida*	AMC, CAZ, FOX, CTX
CBA3	5	*P. paralactis*	AMC, FOX, CTX, K, E
K9	2	*P. paralactis*	AMC, FOX, CTX, MEM, K, E
K14	2	*P. paralactis*	AMC, FOX, CTX, K, E
K19	5	*P. paralactis*	AMC, FOX, CTX, K, E
K36	20	*P. paralactis*	AMC, FOX, CTX, K, E
K44	20	*P. paralactis*	AMC, FOX, CTX, MEM, C, E
CF9	2	*P. paralactis*	AMC, FOX, CTX, MEM, E
CF11	2	*P. paralactis*	AMC, FOX, CTX, E
CF13	2	*P. paralactis*	AMC, FOX, CTX, MEM, C, E
CF15	2	*P. paralactis*	AMC, FOX, CTX, MEM, E
CF20	5	*P. paralactis*	AMC, FOX, CTX, MEM, E
CI2	0	*P. paralactis*	AMC, FOX, CTX, MEM, K, E
CI6	0	*P. paralactis*	AMC, FOX, CTX, K
CI10	2	*P. paralactis*	AMC, CAZ, FOX, CTX, K, S3, E
CI15	2	*P. paralactis*	AMC, FOX, CTX, K, E
CI18	5	*P. paralactis*	AMC, FOX, CTX, K, E
CI20	5	*P. paralactis*	AMC, FOX, CTX, K, E
CI28	10	*P. paralactis*	AMC, FOX, CTX, MEM, E
CI5	0	*P. trivialis*	AMC, CAZ, FOX, CTX, E

* AMC, amoxicillin–clavulanic acid; FOX, cefoxitin; CTX, cefotaxime; CAZ, ceftazidime; MEM, meropenem; CIP, ciprofloxacin; K, kanamycin; E, erythromycin; C, chloramphenicol; S3, sulfonamide.

## Data Availability

Data is contained within the article and supplementary material.

## Supplementary Materials

The following supporting information can be downloaded at: https://www.mdpi.com/article/10.3390/foods11172603/s1, Table S1: Number of reads and alpha diversity indexes at genus level of dressing samples treated or not by high-hydrostatic pressure (HP).
