# Supplementary material for: Antimicrobial Resistance, Biocide Tolerance, and Bacterial Diversity of a Dressing Made from Coriander and Parsley after Application of Treatments Using High Hydrostatic Pressure Alone or in Combination with Moderate Heat
Source: Foods. 2022 Aug 27;11(17):2603. doi: 10.3390/foods11172603 (PMC9455834; doi:10.3390/foods11172603)
Supplement: Supplementary file 1 [file foods-11-02603-s001.zip › foods-1806073-supplementary.pdf]

Supplementary Table S1. Number of reads and alpha diversity indexes at genus level of dressing samples treated or not by high-hydrostatic pressure (HP).

Sample	No. Reads	Chao1	Shannon	Simpson
**U0**	56912	101	2.59	0.82
**U2**	57200	64	1.63	0.65
**U5**	51266	52	1.69	0.74
**U10**	56475	64	1.71	0.72
**U20**	50928	25	0.99	0.45
**A0**	38169	97	3.16	0.93
**A2**	50607	119	3.02	0.91
**A5**	62467	128	3.29	0.93
**A10**	36648	123	3.27	0.93
**A20**	43370	117	3.27	0.93
**B0**	46673	108	3.05	0.92
**B2**	47933	106	3.03	0.91
**B5**	45995	113	3.11	0.92
**B10**	41491	124	3.20	0.93
**B20**	41592	125	3.33	0.94
**C0**	47285	84	2.76	0.88
**C2**	48422	106	2.94	0.88
**C5**	35034	101	3.08	0.91
**C10**	46524	129	3.30	0.93
**C20**	47399	125	3.32	0.92
**D0**	41706	98	2.67	0.87
**D2**	49617	121	3.20	0.93
D5	51462	112	2.98	0.90
D10	41697	116	2.99	0.89
D20	48628	122	3.24	0.91

The U samples correspond to the untreated controls. The different APH treatments are: A, 450 MPa at 22 °C; B, 450 MPa at 50 °C; C, 600 MPa at 22 °C; and D, 600 MPa at 50 °C. The numbers indicate the storage time (days). Chao 1 is an abundance-based estimator of species richness. The Shannon and Simpson indices are estimators of both species richness and evenness, with more emphasis on richness (Shannon) or evenness (Simpson).
